# Feasibility of Remediation of Heavy-Metal-Contaminated Marine Dredged Sediments by Active Capping with *Enteromorpha* Biochar

**DOI:** 10.3390/ijerph19094944

**Published:** 2022-04-19

**Authors:** Zhaowei Wang, Shuang Song, Huan Wang, Wenchao Yang, Jianbo Han, Hong Chen

**Affiliations:** 1 College of Environmental Science and Engineering, Dalian Maritime University, Dalian 116026, China; wzw1128@dlmu.edu.cn (Z.W.); huanw@dlmu.edu.cn (H.W.); 2 Marine Engineering Environmental Supervision Technology Room, National Marine Environmental Monitoring Center, Dalian 116023, China; ssong@nmemc.org.cn (S.S.); jbhan@nmemc.org.cn (J.H.); hchen@nmemc.org.cn (H.C.)

**Keywords:** capping remediation, *Enteromorpha* biochar, dredged sediments, heavy metals

## Abstract

*Enteromorpha* biochar (BC) has been proposed as a potential absorbent in the marine environments. This study attempts to understand the process of active capping using *Enteromorpha* BC to prevent the release of heavy metals (Pb and Cd) from contaminated marine dredged sediments. The capping efficiency was assessed with a series of lab-scale column experiments. Results showed that the *Enteromorpha* BC exhibits rough pore structure and higher specific surface area, as well as more surface organic functional groups, which is favorable for its adsorption capacity and selectivity towards heavy metals. The capping thickness of 2 cm for *Enteromorpha* BC was sufficient to prevent the release of heavy metals from sediments, with the capping efficiency of 47% for Pb and 62% for Cd. Kinetic studies showed that heavy metals released into the overlying water can be described by a three-parameter sigmoidal kinetic model. Importantly, the fractions of heavy metals in the dredged sediments below the capping layer were analyzed to reveal the capping remediation mechanism. The outcomes of the present study indicate that capping with *Enteromorpha* BC is a promising method to regulate the water environment by preventing the release of heavy metals from the contaminated dredged sediments.

## 1. Introduction

Dumping of solid and liquid waste from human activities has resulted in the deposition of large number of harmful substances into the coastal marine systems. These pollutants are deposited into sediments at nearshore and harbor regions, and they maintain a dynamic equilibrium of adsorption and desorption at the sediment–water interface [1]. The resuspension of pollutants in sediments is affected by hydrodynamic conditions and redox conditions, which could further cause a series of environmental and ecological issues in the ocean [2,3]. Heavy metals are typical pollutants in dredged sediments, unlike organic pollutants, which cannot be decomposed by microorganisms but can be enriched in organisms [4,5]. With an increase in awareness of ocean environment protection, the input of heavy metals into marine environments has been regulated. As a result, research foci have shifted to the release of heavy metals from contaminated sediments into marine environments [6,7].

Many measures to control the contaminated sediments are reported in the literature during recent decades, including dredging [8], electrochemical remediation [9], phytoremediation [10], and capping [3,11]. The active capping technology with cleaning materials or active sorbents can degrade the mobility and bioavailability of sedimented heavy metals [12], which has developed rapid and successful cases abroad [13,14]. Soil, coal ash, zeolite, industrial byproducts and activated carbon were used for capping materials research [15,16,17,18]. However, the aforementioned materials are not the most effective considering their limited efficiency, lack of long-term stability, and adverse environmental effects. Therefore, an environmentally friendly capping material that can not only adsorb and immobilize heavy metals but also inhibit the re-suspension of heavy metals for the long-term is needed.

Biochar (BC) is a kind of porous carbon processed from organic waste, which is a stable carbon-rich product [19]. Biochar has the advantages of unique pore structure, large specific surface area, complex surface-active functional groups, and stable chemical properties. The porous structure of biochar results in increased specific surface energy and provides additional effective sites for the removal of heavy metals [20]. In addition, the surface of biochar contains mineral components, which can improve the adsorption performance and enhance the complexation and co-precipitation of biochar and heavy-metal ions [21]. More importantly, heavy-metal ions are generally positively charged; the abundance of negative charges on the surface of biochar increases its cation exchange capacity and enable good adsorption characteristics for heavy metals [22]. Biochar is a promising soil/sediment conditioner that can immobilize and diminish the bioavailability of metal cations through physical adsorption, cation exchange, surface complexation, etc. [23,24]. *Enteromorpha* is a type of marine green alga that is associated with green tides. Masses of free-floating *Enteromorpha* were cleaned up onshore and had not been economically and effectively used [25]. The preparation of *Enteromorpha* BC is an important means of disposal of the large quantity of waste *Enteromorpha*, which is of great significance to solving the environmental and ecological problems caused by *Enteromorpha*. Additionally, the *Enteromorpha* BC has abundant organic functional groups (e.g., O–H groups, [26,27]), and it has the potential to be applied as capping material in the remediation of heavy-metal-contaminated sediments [28]. However, there are very limited studies on the efficiency of active capping using *Enteromorpha* BC to inhibit heavy-metal release.

The main objective of this study was to use the *Enteromorpha*-derived BC as an effective capping material to remediate and dispose of heavy-metal-contaminated dredged sediments in ports. The capping efficiency was assessed with a series of lab-scale column experiments for 45 days at various capping thicknesses. The characteristics of *Enteromorpha* BC, heavy-metal concentrations (Pb and Cd) in the overlying water, and heavy-metal fractions in the capping sediments were analyzed. The novelty in this study is that we focused on the impact of capping thickness on the capping efficiency and the speciation redistribution of heavy metals in capping sediments to understand the capping remediation mechanism. The results provide technical support for the comprehensive treatment of contaminated dredged sediments in ports.

## 2. Materials and Methods

### 2.1. Preparation of Enteromorpha BC and Contaminated Dredged Sediments

*Enteromorpha* powder was purchased from Qingdao Haida Biological Group Co., Ltd., (Qingdao, China). The *Enteromorpha* powder was pyrolyzed in a muffle furnace (KSF-8-11, Yixing Qianjin Furnace Equipment Co., Ltd., Yixing, China) with the temperature rising up to 500 °C from room temperature with a heating rate of 10 °C/min. The *Enteromorpha* BC was produced by stable pyrolysis of the *Enteromorpha* powder at 500 °C for 2 h. Then, the *Enteromorpha* BC was taken out of the furnace after it was cooled down to room temperature (~25 °C). The obtained *Enteromorpha* BC was washed sequentially with 0.5 M HCl and Milli-Q water to neutralize pH (this process may be repeated 3–5 times). Last, the *Enteromorpha* BC was dried at 55 °C for later use.

Dredged sediments that met evaluation criteria for clean dredged materials were transported from the sampling site, the dredging area of Yingkou Port (Yingkou, China, 40.29° N, 122.10° E), and stored in a cool place. Lead (Pb) and cadmium (Cd) were selected as typical target heavy-metal contamination in dredged sediments. To contaminate the dredged sediments, a heavy-metal-mixture solution (the medium was artificial seawater) containing 1000 mg/L of Pb^2+^ and 30 mg/L of Cd^2+^ was prepared and mixed with the dredged sediments in a dark location. The artificial seawater was prepared using NaCl dissolved in Milli-Q water with a salinity of 30. The contamination process lasted for 60 days to obtain the heavy-metal-contaminated dredged sediments, and then the contaminated dredged sediments were dried at 55 °C for later use.

### 2.2. Incubation Experiments

**Figure 1:**
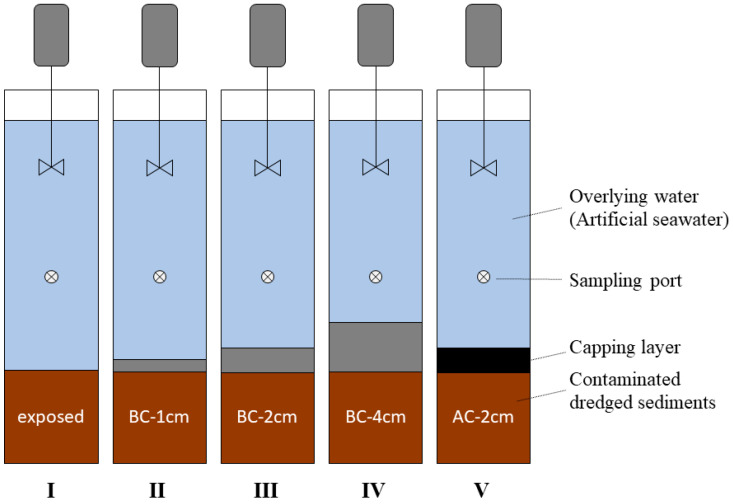
Schematic diagram of the capping experimental set-up.

The laboratory incubation experiments were conducted using lab-made reaction columns to evaluate the capping efficiency with *Enteromorpha* BC (Figure 1). Contaminated dredged sediments (800 g, ~8 cm height inside the column) were disposed uniformly on the bottom of the experimental columns for simulating capping remediation. Consequently, the BC and activated carbon (AC) were uniformly placed on the surface of the contaminated dredged materials with different thicknesses (1, 2, and 4 cm). Finally, 3 L of sampled water (artificial seawater) were injected into each column. Those experiments were carried out at room temperature (~25 °C) for 45 days, and the overlying water was disturbed by a stirring rod. The capping experiments were named BC-1 cm, BC-2 cm, and BC-4 cm for the 1-, 2-, and 4-cm caps of *Enteromorpha* BC, respectively, and AC-2 cm for the 2-cm cap of the commercial AC. No capping was also studied for background and comparative analysis (exposed group).

A sampling port was located 10 cm above the sediment layer at each column (column internal diameter of 12 cm and height of 40 cm). The overlying water (30 mL) was sampled through the sampling port at 0-, 1-, 3-, 5-, 7-, 9-, 15-, 21-, 25-, 32-, 40-, and 45-day intervals after starting the experiment and was analyzed to measure concentrations of heavy metals, including Pb and Cd, to study the capping effect. After collection, samples were filtered with a 0.45-μm filter membrane (Polyethersulfone, Jinteng, Tianjin, China) using a 50-mL syringe. All samples were stored at 4 °C in a dark location until tested. The laboratory incubation experiments were carried out in duplicates to improve the statistic confidence of the experimental data.

### 2.3. Analysis

A scanning electron microscope (SEM, XL-30, Philips, Eindhoven, The Netherlands) was used to observe the surface morphology and structural characteristics of the *Enteromorpha* BC. The Brunner−Emmet−Teller (BET, Autosorb-iQ, Quantachrome, Boynton Beach, FL, USA) method was used to calculate the total specific surface area of the *Enteromorpha* BC sample, and the BJH method was used to calculate the pore diameter and pore volume of the *Enteromorpha* BC. The N_2_ adsorption–desorption experiment was used to determine the specific surface area and pore size distribution of *Enteromorpha* BC at 77 K. Fourier transform infrared (FT-IR) spectroscopy (Nicolet IR200, Thermo Electron, Waltham, MA, USA) was used to analyze the types of organic functional groups on the surface of *Enteromorpha* BC (resolution: 2 cm^−1^, scanning speed: 4.00 cm/s, scanning interval: 400–4000 cm^−1^).

Sequential extraction and quantification of heavy metals. The heavy-metal fraction in the initial contaminated dredged sediments and the sediments below the capping layer after the incubation experiments were analyzed by improved BCR extraction methods [29]. Each fraction and its procurement method were the following: (1) exchangeable state (F1), 40 mL 0.11 mol/L HOAC was added to 1 g of the sediment sample and centrifuged at 150 rmp for 16 h; (2) reducible state (F2), 40 mL 0.5 mol/L NH_2_OH·HCl was added to the sample (acidification with HNO_3_ to pH = 2) and centrifuged at 150 rmp for 16 h; (3) oxidizable state (F3), 10 mL H_2_O_2_ (pH = 2–3) was added to the sample and allowed to react at room temperature for 1 h before heating to (85 ± 2) °C and reacted for 1 h; then 40 mL 1 mol/L NH_4_OAC (acidification with HNO_3_ to pH = 2) was added after the removal of the H_2_O_2_ hydrogen peroxide and centrifuged at 150 rmp for 16 h; (4) residual state (F4), 6 mL HCl, 4 mL HNO_3_, 8 mL HF, and 1 mL HClO_4_ were added to the residual and digested with a fully automatic graphite digester (GDANA, Guangzhou, China, DS-72). All reagents used in the experiments were of guaranteed grade. An atomic absorption spectrophotometer (graphite furnace method, Perkin Elmer, 4110ZL, San Francisco, CA, USA) was used to determine the concentration of heavy metals in overlying waters and digestion solutions. The detection limit was 1 µg/L, stability ≤ 1.0% at 2 h, and precision ≤ 1.0%.

## 3. Results and Discussion

### 3.1. Characterization of Enteromorpha BC

**Figure 2:**
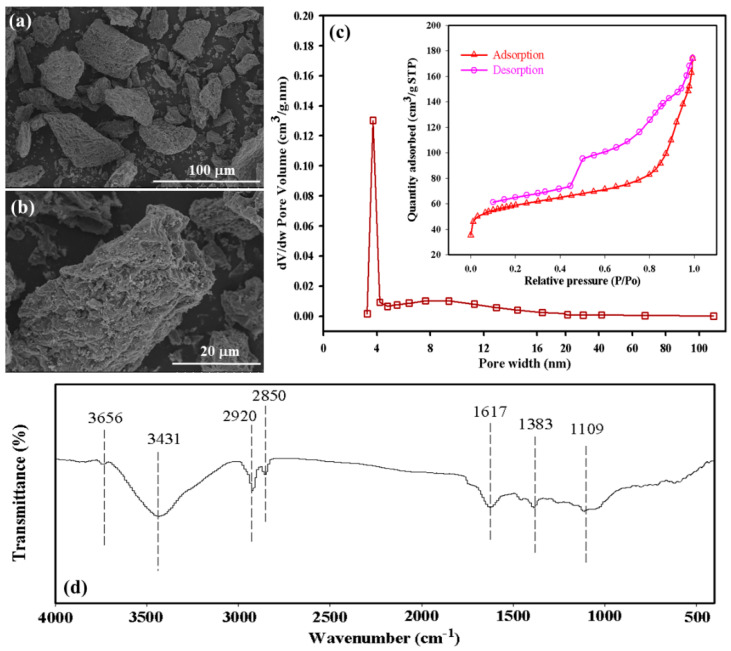
(**a**,**b**) SEM image of BC, (**c**) pore size distributions and N_2_ adsorption–desorption isotherms of BC, and (**d**) FT–IR spectra of BC.

*Enteromorpha* BC is used as a capping material to regulate the water environment by preventing the release of heavy metals from sediments. Pore structure including surface area, porosity, and pore size are the important physical properties of BC and are a important factors in controlling the mobilization of pollutants by BC [25,30]. The SEM results of the *Enteromorpha* BC properties are shown in Figure 2a,b. The surface of the *Enteromorpha* BC displays different depths and irregularly arranged foam cell structures. In addition, a rough pore structure is documented on the surface of *Enteromorpha* BC. The porous structure of *Enteromorpha* BC results in an increase in its specific surface area and adsorption sites, which is favorable for its adsorption capacity and selectivity towards contaminants [31]. The study of the pore characteristics of *Enteromorpha* BC is based on the 77K N_2_ adsorption–desorption experiment (Figure 2c). The *Enteromorpha* BC are micro-, meso-, and macro-porous coexisting materials, with meso-porous materials comprising the majority. The BET specific surface area of the *Enteromorpha* BC is 214.8 m^2^ g^−1^. The pore volume and pore diameter are 0.26 cm^3^/g and 7.1 nm, respectively.

Carboxyl groups are known to facilitate the removal of heavy metals from contaminated sediments [26]. FT-IR spectroscopy results (Figure 2d) show that the surfaces of *Enteromorpha* BC are rich in organic functional groups. In the figure, the absorption peak near 3431 cm^−1^ corresponds to the asymmetric stretching vibration of –OH in alcohols, phenols, or carboxyl groups. The absorption peak near 2920 cm^−1^ corresponds to C–H vibration, and the absorption peak near 1617 cm^−1^ corresponds to –COOH vibration. The change between 1660^−1^ and 1500 cm^−1^ could be attributed to the C=C stretching vibration, which is related to the aromatic C–C bond and the asymmetric-COOH stretching. The absorption peak at 1109 cm^−1^ corresponds to the C–O stretching vibration of carboxyl or esters. Most of the FTIR peaks are from organic functional groups commonly observed in *Enteromorpha* BC, such as the carboxyl and hydroxyl groups, which exhibit good affinity towards metal cations, and those functional groups can also interact with various heavy metals by forming complexities [32]. In addition, the aromatic carbon can provide π-electron, which was reported to have potential to bond with heavy-metal cations [33]. Our results show that the *Enteromorpha* BC is rich in organic functional groups, which could be a promising and effective capping material in remediating contaminated dredged sediments.

### 3.2. Effect of Capping on the Release of Heavy Metals from Dredged Sediments

BC capping can effectively reduce the release of pollutants from sediments, and the capping thickness is the most effective factor to control the pollutants flux across the sediment–water interface [34]. This study focuses on the heavy-metal concentration in the overlying water to understand the effect of capping treatment. The capping efficiency was calculated by Equation (1) [35]:

(1) $$P\;\left(\%\right)=\frac{C_{e,\;t}-C_{t}}{C_{e,t}}\times 100\%,$$

where *P* (%) is the capping efficiency, *C_e,t_* (mg/L) is the concentration of heavy metals in overlying water in the exposed group, and *C_t_* (mg/L) is the concentration of heavy metals in overlying water in the capping groups.

**Figure 3:**
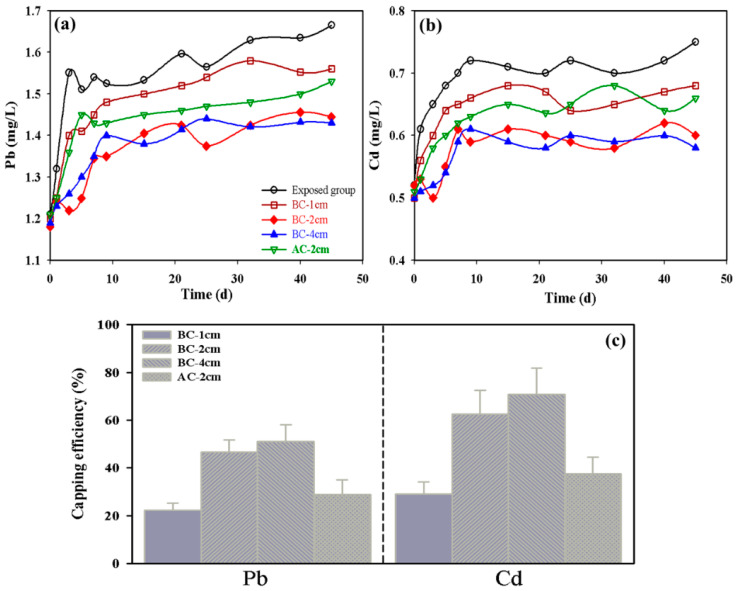
(**a**,**b**) Concentrations of heavy metals in the overlying water during the capping experiments and (**c**) the capping efficiency by BC.

The results of capping thickness on the release of Pb and Cd from contaminated dredged sediments is shown in Figure 3a,b. The concentration of Pb and Cd in the overlying water during the initial time was 1.20 mg/L and 0.50 mg/L, respectively. Under uncapped conditions, the Pb and Cd concentrations increased sharply within the first three days followed by a slow increase. The final concentrations for the exposed group converged at 1.66 mg/L for Pb and 0.75 mg/L for Cd. The change of Pb concentration during the experimental run in the BC-1 cm, BC-2 cm, BC-4 cm, and activated carbon-2 cm capping increased from 1.20 mg/L to 1.56 mg/L, 1.44 mg/L, 1.43 mg/L, and 1.53 mg/L, respectively. The Cd concentration increased from 0.50 mg/L to 0.68 mg/L, 0.60 mg/L, 0.58 mg/L, and 0.66 mg/L, respectively. The release of heavy metals from sediments capped with *Enteromorpha* BC increased continuously but significantly less than under the uncapped conditions.

The concentrations of heavy metals in the four capping groups were significantly lower than those in the exposed group without capping (*t*-test, *p* < 0.05), indicating that capping treatments efficiently blocked the release of heavy metals. However, the BC capping thickness between 2 and 4 cm had no significant effect on the release of heavy metals (*t*-test, *p* < 0.05), which suggests that the *Enteromorpha* BC capping thickness of 2 cm was optimal for preventing the release of heavy metals from sediments. The capping efficiency was calculated from the data and is presented in Table 1 and Figure 3c. Results show that the capping efficiency after 45 d incubation for the BC-1 cm, BC-2 cm, BC-4 cm, and activated carbon-2 cm capping group were 22%, 47%, 51%, and 29% for Pb and 29%, 62%, 70%, and 38% for Cd, respectively. Adsorption of heavy metals on biochar is also dependent on the initial concentrations of the heavy-metal ions [36], and the lower initial concentration of heavy-metal ions may enhance the collision probability between heavy metals and biochar. Therefore, Cd is easier to combine with the adsorption site on *Enteromorpha* BC [37]. Results of this study suggest that the *Enteromorpha* BC is more effective than the AC in capping remediation process. Literature studies have shown that the surface characteristics and chemical properties of carbon are more important factors in determining its ability to adsorb and fix heavy metals. The *Enteromorpha* BC exhibits higher specific surface area and cation exchange capacity, as well as more surface functional groups, which is more favorable for the adsorption of heavy metals compared to AC [20,21,22]. Thus, the *Enteromorpha* BC could be a promising capping material in remediating contaminated sediments, particularly with heavy metals [38].

### 3.3. Heavy-Metal Fraction in Dredged Sediments under the Effect of Capping

**Figure 4:**
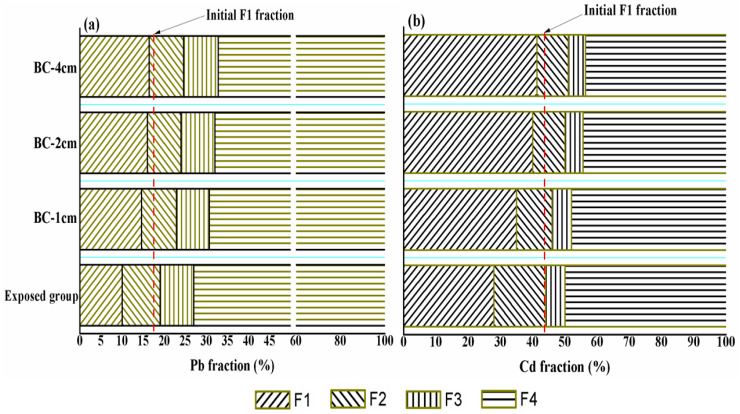
Heavy-metal fraction of Pb (**a**) and Cd (**b**) in dredged sediments by BCR methods.

To specify the effect of capping on mobilization risk of heavy metals in dredged sediments, the fractions of heavy metals in the contaminated dredged sediments before and after the capping treatment were analyzed and are presented in Table 2 and Figure 4. The F1, F2, F3, and F4 fractions in the exposed sediment are presented as 10%, 9%, 8%, and 73% for Pb and 28%, 16%, 6%, and 50% for Cd, respectively. In contrast, the change of fraction of Pb and Cd in the sediments under the BC capping layer is mainly reflected in the speciation of F1. The F1 fractions in the initially contaminated dredged sediments were 17% for Pb and 43% for Cd. Our results show that the speciation of F1 in the sediments of the exposed group decreased to 10% for Pb and 28% for Cd, indicating that the major contribution of heavy metals diffused into the overlying water were in the exchangeable state [39,40]. The dredged sediments capped with *Enteromorpha* BC possess a high percentage of F1. The speciation of F1 gradually increased following the capping thickness increase from 1 to 4 cm, indicating that the capping treatment could change the speciation redistribution of heavy metals in sediments. Capping with *Enteromorpha* BC has a positive effect on stabilizing the heavy metals in sediments. The organic functional groups (e.g., –COOH, –OH, –NH^4+^, and –C=C–) in *Enteromorpha* BC can affect the speciation distribution of heavy metals and reduce migration activity of heavy metals in sediments through surface precipitation [41,42]. The speciation change of heavy metals in sediments is relatively slow; therefore, the improvement of long-term stability of heavy metals in sediments under the effect of BC capping needs to be investigated in the future.

### 3.4. Time-Scale and Release Kinetics for Capping Treatment

**Figure 5:**
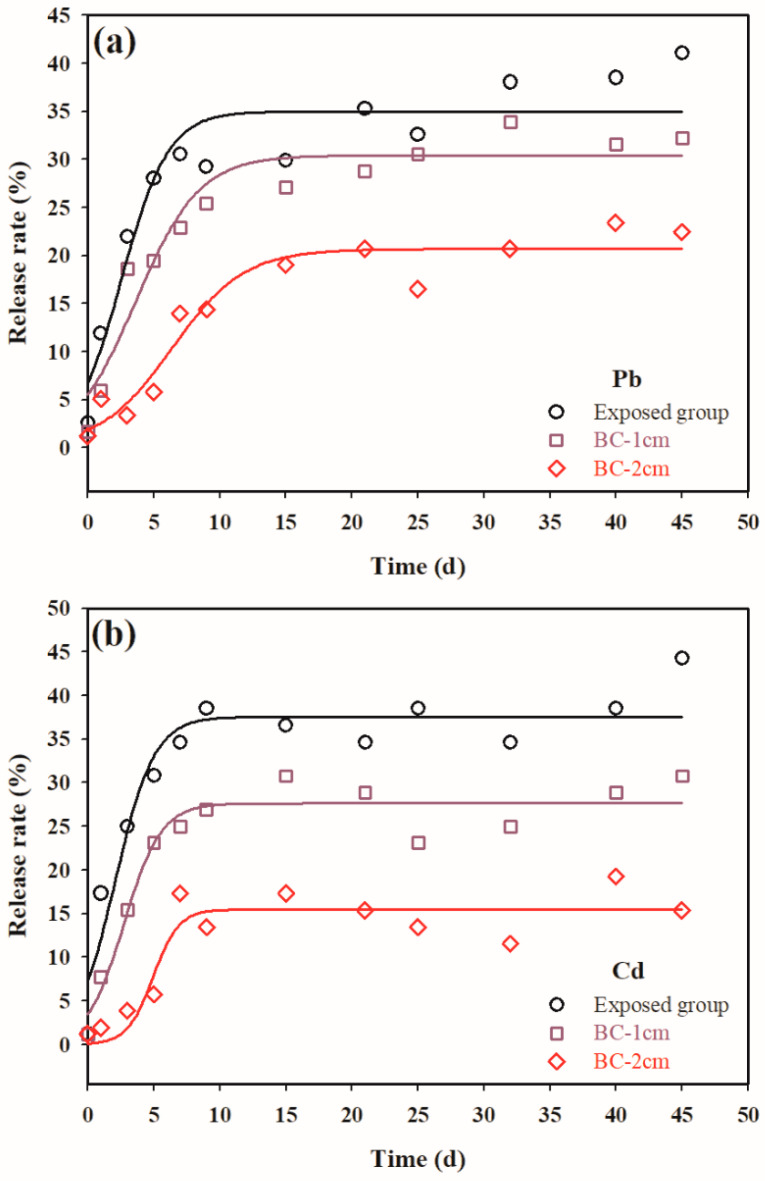
Kinetics of release of Pb (**a**) and Cd (**b**) into the overlying water by BC capping.

Capping remediation of contaminated dredged sediments is a long process [34]. The release kinetics of pollutants from contaminated dredged sediments followed by BC capping treatment is valuable for risk assessment and policymaking. Our experiment simulated the capping treatment. The release rate (Equation (2)) was used to evaluate the kinetics of heavy metals diffusing into the overlying water:

(2) $$R\left(\%\right)=\frac{C_{t}-C_{i}}{C_{i}}\times 100\%$$

where *R* is the release rate of heavy metals, *C_t_* (mg/L) is the concentration of heavy metals in the overlying water at time *t*, and *C_i_* (mg/L) is the concentration of heavy metals in the overlying water during the initial time. In this study, *C_i_* is 1.2 mg/L for Pb and is 0.5 mg/L for Cd. Figure 5 shows the release rate of heavy metals as a function of time (0 to 45 day). The release rate (*R*, %) of heavy metals increased rapidly with the reaction time first and then gradually flattened as equilibrium was reached within 10 d. The variations of *R* with time can be described by Equation (3):

(3) $$R\left(\%\right)=\frac{R_{max}}{1+e^{-\frac{t-t_{0}}{b}}}$$

where *R_max_* is the maximum release rate (%), *t* is the reaction time (day), *t*_0_ is the critical time when *R* change reaches the maximum, and b is a constant that controls the release kinetics curve [43].

The corresponding parameters describing the kinetics of Pb and Cd release from the capping treatment were calculated and summarized in Table 3. Typically, the release of heavy metals from contaminated dredged sediments into overlying water occurs in two consecutive steps [44]: (1) heavy metals dissolve into pore water (see discussion in Section 3.3), and (2) heavy metals diffuse from pore water to overlying water through the capping layer. The effect of the capping layer in blocking the diffusion of heavy metals becomes more significant. Several mathematical models have been developed to describe the kinetics of metals on biochar, e.g., the pseudo-first order, pseudo-second order, Elovich, and intraparticle diffusion model [45]. In this study, we used a three-parameter sigmoidal kinetic model focusing on the diffusive transport of metal ions from marine sediments into the biochar’s active sites and overlying water, which describes interaction rates between biochar and specific metal ions. Our results are consistent with those of Ofomaja [46] and Yang et al. [43], who revealed a rapid removal of heavy metals at the initial time then equilibrium, and those processes depended greatly on the physical and/or chemical characteristics of the biochar. This study proposes that the capping thickness of 2 cm BC can achieve the best blocking effect for Pb and Cd.

The maximum Pb and Cd release rate for the exposed group reached 35% and 37%, respectively, within 45 days, and the maximum increase of the release rate occurred on the third day for Pb and on the second day for Cd. After the capping treatment, the maximum Pb and Cd release rate decreased to 21% and 16%, respectively. The maximum increase rate of the release delay occurred on the sixth day for Pb and on the fifth day for Cd. This dynamic process is another indicator of effectivity of *Enteromorpha* BC in interrupting heavy-metal release. The heavy metals trapped beneath the capping layer are transformed as an easily mobile heavy-metal fraction in sediments [47]. When compared with the heavy metals released from the exposed group, the heavy-metal concentration under the *Enteromorpha* BC capping treatment was reduced by 40% for Pb and 57% for Cd. Additionally, the diffusion of heavy metals was effectively inhibited with the increase of *Enteromorpha* BC dose. Our results indicate that the *Enteromorpha* BC capping played an important role in inhibiting heavy-metal release as indicated by the increase in the heavy-metal mobile fraction in sediments. Although the *Enteromorpha* BC has its advantages in adsorbing and stabilizing heavy metals, but it is relatively difficult to apply in practical engineering cases due to its light specific gravity and easy disturbance under typical marine dynamic conditions. In the future, we may consider mixing the *Enteromorpha* BC into the solidified materials, and then perform in situ capping remediation of contaminated dredged sediments in ports.

## 4. Conclusions

In this study, environmentally benign BC derived from *Enteromorpha* was used to remediate and dispose of heavy-metal-contaminated dredged sediments using the capping method. The novelty in this study is that we focused on the impact of capping thickness on the capping efficiency and the speciation redistribution of heavy metals in capping sediments to understand the capping remediation mechanism. The results suggest that *Enteromorpha* BC is a promising capping material in remediating heavy-metal-contaminated dredged sediments, but it is relatively difficult to apply in practical engineering cases due to its light specific gravity and easy disturbance under typical marine dynamic conditions. In the future, it can be considered to mix the *Enteromorpha* BC into the solidified materials, and then perform in situ capping remediation of contaminated dredged sediments in ports.

## Figures and Tables

**Table 1:** Effect of capping thickness on the capping efficiency by *Enteromorpha* BC.

Capping Thickness	Capping Efficiency (%)	
	Pb	Cd
1 cm	22.2 ± 3.0	29.1 ± 4.8
2 cm	46.7 ± 5.1	62.5 ± 9.9
4 cm	51.1 ± 6.9	70.8 ± 11.1

**Table 2:** Speciation redistribution of heavy metals in sediments.

Speciation	Pb				Cd			
	Exposed	BC-1 cm	BC-2 cm	BC-4 cm	Exposed	BC-1 cm	BC-2 cm	BC-4 cm
F1 (%)	10.0	14.6	16.0	16.4	28.0	35.0	40.0	41.3
F2 (%)	9.0	8.3	8.0	8.2	16.0	11.0	10.0	9.7
F3 (%)	8.0	7.8	8.0	8.2	6.0	6.0	5.6	5.4
F4 (%)	73.0	69.3	68	67.2	50.0	48.0	44.4	43.6

**Table 3:** Kinetic model parameters describing the release of Pb and Cd from the capping treatment.

		*R_max_* (%)	*t*_0_ (d)	b	*r* ^2^
**Pb**	Exposed group	35	3	1.7	0.8991
	BC-1 cm	30	4	2.4	0.9300
	BC-2 cm	21	6	2.7	0.9334
**Cd**	Exposed group	37	2	1.5	0.9092
	BC-1 cm	28	3	1.4	0.9387
	BC-2 cm	16	5	1.0	0.8563

## Data Availability

The data for this study are available from the corresponding author via email: wcyang@nmemc.org.cn.
